# Genetic and environmental regulation of early heading in photoperiod-insensitive rice: impacts on heading synchrony, premature heading, and tiller development lag

**DOI:** 10.1270/jsbbs.25002

**Published:** 2025-07-31

**Authors:** Shuntaro Sakaguchi, Yuya Ota, Md. Imdadul Hoque, Yuji Kishima

**Affiliations:** 1 Research Faculty of Agriculture, Hokkaido University, Kita-9 Nishi-9, Kita-ku, Sapporo, Hokkaido 060-8589, Japan; 2 Mie Prefecture Agricultural Research Institute, 530 Ureshinokawakita, Matsusaka, Mie 515-2316, Japan

**Keywords:** early heading, effective tillering, heading synchrony, photo-insensitive, premature heading, rice, QTL

## Abstract

The process by which day length affects rice heading has been studied on a molecular basis; however, little is known about the traits that occur with heading, such as heading synchrony, premature heading, and day of the determined effective tiller number. These traits vary more in photo-insensitive (photo-In) lines than in day-length-sensitive lines. We used photo-in lines to study the associations of these traits with heading earliness and the influence of the earliness genes and the different latitudes. The results of this study showed these traits were significantly correlated with the earliness of first days to heading (F-DTH) among the photo-In lines. A wider range of F-DTH was observed in Sapporo than Iga when used the same photo-In varieties, which was confirmed under artificial conditions. We identified five significant quantitative trait loci (QTL) for F-DTH among the photo-In lines. A QTL with a major effect associated with F-DTH, heading synchrony, and premature heading was detected on chromosome 4. Using photo-In lines, we propose a heat-tolerance strategy for early cultivation in low-latitude regions.

## Introduction

Heading date is the key factor that determines the regional adaptability of rice (*Oryza* *sativa* L.) (Hori *et al.* 2016, Nonoue *et al.* 2008). Recently, high temperatures caused by global warming have led to heat stress, which is expected to reduce rice yield (Zhao *et al.* 2017). One way to avoid stress is to adjust the growing season by introducing early- or late-heading varieties (Chen *et al.* 2018, Nemoto *et al.* 2011). The appropriate timing of flowering is important not only for reproductive success, but also for sufficient vegetative growth to achieve high yields (Fujino 2020, Hu *et al.* 2015). Understanding and selecting varieties with appropriate heading characteristics are necessary to ensure rice production (Chen *et al.* 2018).

**Fig. 1.**
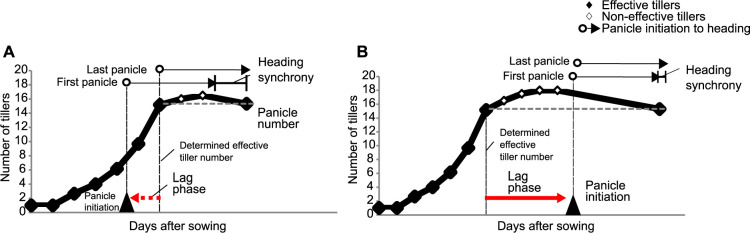
Lag phase pattern based on panicle initiation and day of the determined effective tiller number. Lag phase was calculated as the days to panicle initiation minus day of the determined effective tiller number. Lag phase shows negative values when panicle initiation occurs before the day of the determined effective tiller number (A) and positive values when panicle initiation occurs after the day of the determined effective tiller number (B). In general, early heading lines indicate negative values of lag phase (A) and mid- and late-heading lines indicate positive values of lag phase (B). Black fills indicate effective tillers and white fills indicate non-effective tillers. Early-heading varieties show a higher ratio of effective tillers producing panicles to the total number of tillers than the middle- and late-heading varieties (Takeda 1986).

Many studies define the time of flowering in cereals as the stage when 50% of the tillers have headed or when the first panicle begins to emerge (Lukac *et al.* 2012, Ma *et al.* 2009). However, rice has multiple tillers that appear sequentially, and it takes a considerable number of days from the first to the last panicle heading. This period is called heading synchrony and is an agronomic trait that influences rice yield and grain quality (Takeda 1986; Fig. 1). Earlier-heading panicles are superior to later-heading panicles in terms of panicle weight, filled grain rate, and quality (Counce *et al.* 1996, Nakayama and Saito 1978). Short-term completion of heading panicles is desirable for synchronous maturation to harvest, whereas the simultaneous development of panicles increases environmental constraints such as cold and heat stress (Takeda 1986). Heading synchrony is known to correlate with heading earliness, and early-heading varieties generally show a long period of heading synchrony (Ma *et al.* 2009, Takeda 1986).

Premature heading is a characteristic of rice plants that are extremely-early headed at the same time as development of the tiller and do not allow fully mature panicles to form. Immature panicles resulting from premature heading are the first panicles to form on the main stem and are, therefore, directly related to yield reduction (Hu *et al.* 2015, Osada *et al.* 1975). Photo-insensitive (photo-In) varieties are more prone to premature heading when exposed to high temperatures during the seedling stage, whereas photo-sensitive (photo-Se) varieties are less prone (Hu *et al.* 2015, Osada *et al.* 1975, Sakata *et al.* 2004). However, the genetic relationships between the premature heading and heading earliness have not been fully analyzed.

Tillers that produce panicles are called effective tillers and tillers that do not produce panicles are called non-effective tillers. In the early-heading varieties, each panicle development occurs simultaneously with tiller development, and effective tillers are gradually produced as tillers form. In contrast, in the middle- and late-heading varieties, panicle development is likely to occur in the developed tillers, and most effective tillers initiate panicles at similar times. The period from day of the determined effective tiller number to panicle initiation is called the lag phase and an important trait related to heading synchrony (Takeda 1986; Fig. 1). Early-heading varieties have negative values for the lag phase because panicle initiation precedes the determination of the number of effective tillers. For middle- and late-heading varieties, the lag phase usually show positive values (Takeda 1986). When premature heading occurs, the lag phase has a large negative value and panicle initiation of the main stem occurs much earlier than in tillering.

Early-heading varieties of rice usually lack photo-sensitivity and initiate panicle development, even under long day lengths (Sakaguchi *et al.* 2024). In high latitude regions such as Sapporo, Hokkaido, Japan (>43° N), the day length of more than 13 h continues from early April to early September and the average ambient temperature during this period is 16°C. The extreme natural conditions in the high-latitude region to which the early-heading varieties have adapted may influence not only the heading date but also the heading characteristics, including days to panicle initiation, heading synchrony, days of the determined effective tiller number, and lag phase. Compared to the conditions in the lower-latitude region, the responses of the early-heading varieties in the higher-latitude region may provide different heading characteristics influenced by photo-insensitivity.

In this study, we compared heading traits such as heading synchrony, premature heading, and effective tillering in the photo-Se and photo-In varieties at different latitudes, Sapporo and Iga, Mie, Japan (34° N). The relationships between these heading traits and heading earliness were elucidated to understand the reproductive adaptation of early heading rice varieties. We attempted to detect quantitative trait loci (QTL) for these traits in the recombinant inbred lines (RILs) using the genome-wide SNP markers (Koide *et al.* 2019). These results not only contribute to the understanding of the genetic basis of heading traits, but also to the application of the photo-In varieties to avoid the intense heat summer.

## Materials and Methods

### Plant materials and growth conditions

The experiments were conducted in two consecutive years (2018 and 2019) in two different paddy fields: one at Hokkaido University, Sapporo, Japan (43.1° N) and the other at Mie Prefectural Agricultural Research Institute, Iga, Japan (34.4° N). Seeds were sown in early May and late April in Sapporo and Iga, respectively, and three- to four-week-old plants were transplanted into paddy fields (Table 1). Six plants of each variety were examined.

The materials used in this study are listed in Table 1. In 2018, we used two photo-In varieties adapted to Hokkaido, A58 and Kitaake, and the 30 RILs derived from a cross between A58 and Kitaake. Thirty RILs consisting of 15 each of early- and late-heading lines were selected from 132 lines in the F3 generation (Koide *et al.* 2019). We selected two of the earliest heading RILs (E-RIL-1 and -2) and two of the latest heading RILs (L-RIL-1 and -2) in 2018. In 2019, we evaluated four RILs (E-RIL-1, -2 and L-RIL-1, -2) and nine photo-In varieties (A58, Kitaake, Akage, Norin 20, Hakuchomochi, Kirara 397, Hoshinoyume, Nanatsuboshi, and Daichinohoshi), which were divided into six groups of rice populations in Hokkaido based on their genomic structure (Shinada *et al.* 2014). As the photo-Se variety, we used Koshihikari in all the test plots, and Mie 33 in Iga for two years and in Sapporo in 2019.

Four photoperiod sensitivity genes (*E1/Hd4/Ghd7*, *Hd2/OsPRR37*, *Se1/Hd1*, and *Hd5/DTH8*) strongly contribute to the adaptation of rice to high-latitude areas, Hokkaido (Fujino and Sekiguchi 2005a, 2005b, Fujino *et al.* 2013, Gao *et al.* 2014, Ichitani *et al.* 1997, Koo *et al.* 2013). The genotypes of these four genes in the materials used in this study are shown in Supplemental Table 1. *Ghd7* and *Osprr37* are fixed for non-functional alleles and *Hd1* and *DTH8* genotypes varied among materials (Fujino *et al.* 2019). A58/Kitaake RILs have either non-functional (A58 type) or functional (Kitaake type) allele (Koide *et al.* 2019).

### Heading properties analysis

The days to heading (DTH) of all tillers on each plant were recorded. The heading status of each plant was designated as the first days to heading (F-DTH), days from sowing to first panicle emergence, last days to heading (L-DTH), and days from sowing to last panicle emergence. The panicle number per plant was defined as the number of panicles headed from the F-DTH to the L-DTH. Heading synchrony and the degree of premature heading (Deg_Pre_Hed) were calculated using the following equations:

Heading synchrony = (L-DTH) – (F-DTH)

Deg_Pre_Hed = (days from sowing to second panicle heading) – (F-DTH)

We calculated the lag phases of A58, Kitaake, four RILs (E-RIL-1, -2 and L-RIL-1, -2), and Koshihikari. Lag phase was calculated by subtracting the days to panicle initiation from the day of the determined effective tiller number.

The number of tillers was recorded every week, starting three weeks after transplanting, and the average tiller number for each variety was calculated. The day of the determined effective tiller number for each plant was assessed as the number of days from sowing until the number of tillers reached the panicle number (Fig. 1).

To estimate the time of panicle initiation in the paddy fields, we grew the same materials in a greenhouse. We examined the periods from the panicle initiation to heading using the greenhouse materials, and subtracted the examined periods from the F-DTH of the paddy field to predict the date of panicle initiation in the paddy field. The panicle initiation date of the rice materials grown in greenhouse was determined according to Sakaguchi *et al.* (2024).

### Correlation analysis

The correlation coefficients among F-DTH, L-DTH, heading synchrony, Deg_Pre_Hed, and panicle number for each experimental condition were calculated using Excel 2019 (Microsoft). The correlation coefficients between years and locations were estimated using Excel 2019.

### QTL analysis

QTL analysis of heading traits and panicle number were performed for the 30 RILs. Previously, 634 markers from 30 RILs were detected using ddRAD-seq (Baird *et al.* 2008, Koide *et al.* 2019, Peterson *et al.* 2012). For QTL analysis, among the 634 polymorphisms, we selected 224 polymorphisms that were confirmed by the A58 whole-genome resequencing data and the Kitaake sequence from the DDBJ database (accession numbers DRA007777 and SRA054074, respectively). A linkage map was constructed using version 3.0 of MAPMAKER/EXP (Lander *et al.* 1987). QTL analysis was performed by composite interval mapping using Windows QTL Cartographer v.2.5 software (Wang *et al.* 2012). The analysis parameters were standard model 6, window size of 10 cM, walking speed of 1 cM, and forward and backward regression models. A significance threshold value (α = 0.05) was determined with 1000 permutations for each trait. Multiple-trait composite interval mapping was also performed to detect pleiotropic QTL and to test the presence of QTL by environmental interaction.

### Photoperiod and temperature sensitivity analysis

A58, Kitaake, E-RIL-1, -2 and L-RIL-1, -2 were examined for F-DTH. Each line consisted of four plants. Plants were grown in growth chambers under four different controlled environments, which comprised combinations of two photoperiod conditions (14 h light/10 h dark and 12 h light/12 h dark) and two temperature conditions (25°C light/20°C dark and 22°C light/18°C dark). Tillers were removed when they emerged.

## Results

### Environmental differences between Sapporo and Iga

**Fig. 2.**
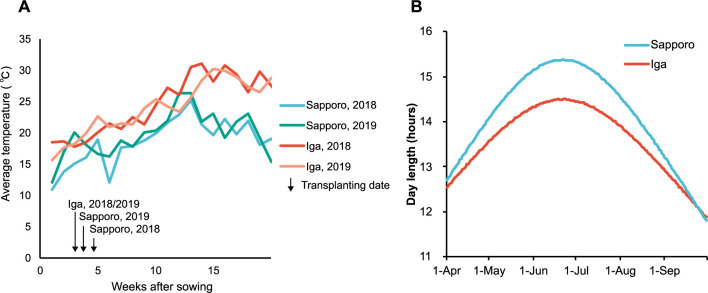
Environmental conditions in Sapporo and Iga. (A) Average weekly temperature obtained from the stations of AMEDAS. Arrows indicate the transplanting date of each experimental plot. (B) Day length condition from April to the end of September.

To investigate the environmental effects on heading characteristics, experiments were conducted in two years in two different paddy fields in Sapporo (43° N) and Iga (34° N). The sowing and transplanting dates were set at each site according to the local growing season (Table 1). The seedling periods were 32 and 26 days in Sapporo in 2018 and 2019, respectively, and 21 days in Iga in both years. In both 2018 and 2019, the temperatures were generally lower in Sapporo than in Iga (Fig. 2A). However, due to the longer seedling periods in Sapporo, the accumulated temperature until transplanting was higher in Sapporo (two-year average: 476°C) than in Iga (two-year average: 389°C). The difference between the two latitudes resulted in longer daylight hours at Sapporo than at Iga throughout the growing season (Fig. 2B).

### Variations of F-DTH, L-DTH, heading synchrony, and Deg_Pre_Hed in Sapporo and Iga

**Fig. 3.**
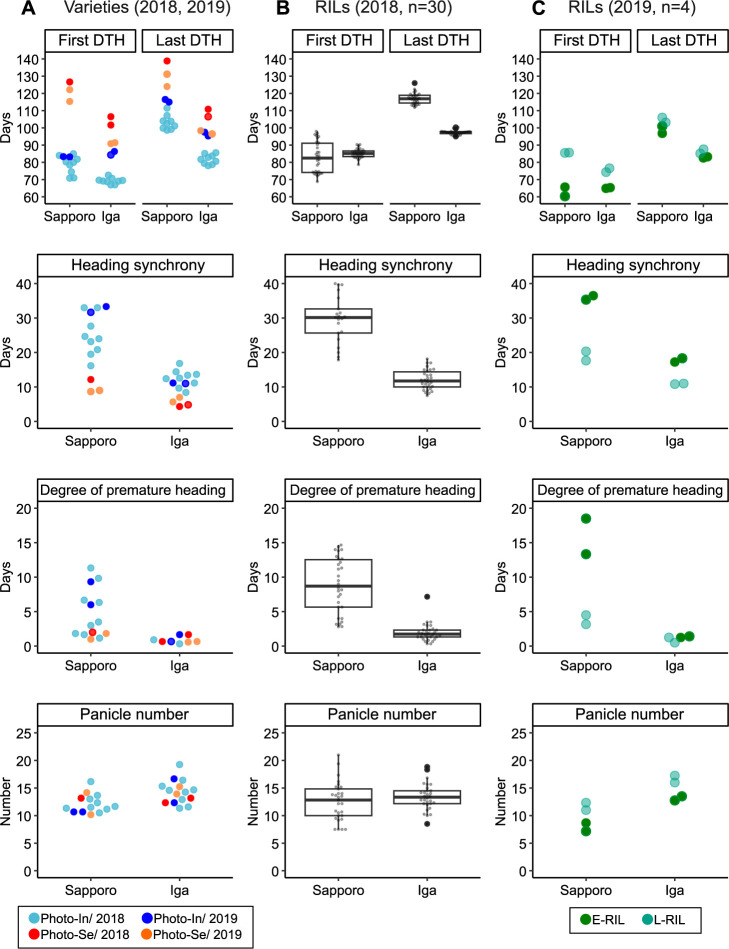
Variations of four heading traits and panicle number in Sapporo and Iga. (A) The materials used in 2018 and 2019 from the photo-In and -Se varieties were common to Sapporo and Iga, listed in Table 1. (B) In 2018, the materials were derived from the 30 RILs were used in both Sapporo and Iga. (C) In 2019, the four materials of two early and late RILs (E-RIL-1, -2 and L-RIL-1, -2) were tested in both Sapporo and Iga. The first DTH, last DTH, heading synchrony, degree of premature heading, and panicle number in Sapporo and Iga over the two years are shown. Each dot represents the average of each variety.

Rice varieties adapted to high-latitude areas are photo-In and early heading; however, their heading synchronies are relatively long (Ma *et al.* 2009, Takeda 1986). The two-year averages of F-DTH for photo-In varieties were 79 and 72 days in Sapporo and Iga, respectively, whereas the F-DTH averages for photo-Se varieties were 121 and 98 days, respectively (Fig. 3A). The difference in F-DTH between the photo-Se and -In lines was greater in Sapporo than in Iga (Fig. 3A), indicating that heading of photo-Se varieties was suppressed by longer day length in Sapporo. Comparing the two-year averages, the differences in F-DTH between photo-In lines and photo-Se varieties were 42 days in Sapporo and 25 days in Iga, while the differences in L-DTH were 26 days in Sapporo and 19 days in Iga. Based on average, the difference in F-DTH was larger than that in L-DTH (Fig. 3A). The variation in L-DTH in Iga was smaller than that in Sapporo, and while L-DTH took about 100 to 140 days in Sapporo, it took only about 80 to 110 days in Iga (Fig. 3A). Taking the above data together, the heading synchrony of the photo-In varieties (26 days in Sapporo and 12 days in Iga on a two-year average) was longer than that of the photo-Se varieties (10 days in Sapporo and five days in Iga on a two-year average) (*t*-test, P < 0.01) (Fig. 3A). Premature heading also exhibited a trend similar to that of heading synchrony. In Sapporo, Deg_Pre_Hed of the photo-Se varieties were less than three days in two years, whereas the photo-In varieties showed Deg_Pre_Hed ranging from 1 to 11 days (Fig. 3A). Although some of the photo-In varieties had Deg_Pre_Hed similar to that of the photo-Se varieties, the photo-In varieties tended to have a longer Deg_Pre_Hed than the photo-Se varieties (Fig. 3A). In Iga, all varieties had two days or less Deg_Pre_Hed in both years, and there were no differences between the photo-Se and -In varieties (Fig. 3A) (*t*-test, P > 0.1). There were no clear differences in panicle numbers between the photo-Se and -In lines (Fig. 3A) (*t*-test, P > 0.1).

### Variations in panicle initiation, effective tiller, and lag phase in Sapporo and Iga

The difference between the time of appearance of tillers that reached the number of effective tillers and the time of panicle initiation was defined as the lag phase (Takeda 1986; Fig. 1). Here, we compared the lag phase of two photo-In varieties (A58 and Kitaake), four RILs (E-RIL-1, -2, and L-RIL-1, -2), and Koshihikari in 2019 in Sapporo and Iga.

**Fig. 4.**
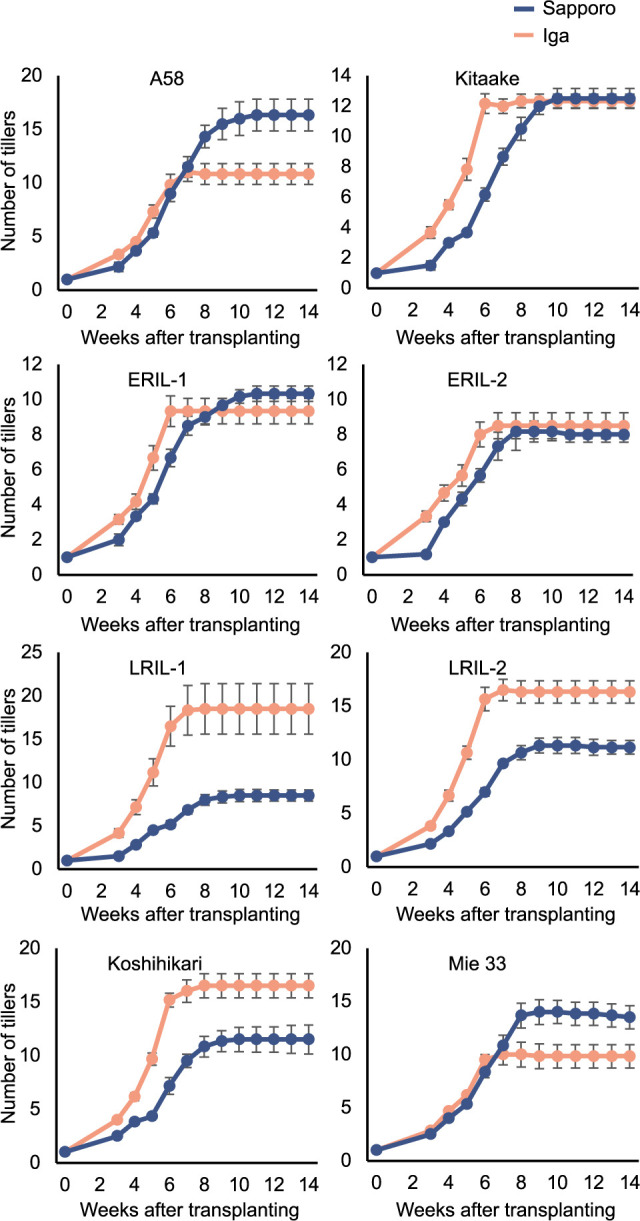
Tiller number transitions of the eight lines in Sapporo and Iga in 2019. Each dot indicates the average number of tillers in Sapporo (blue) and Iga (red). Tiller numbers were recorded every week from three weeks after transplanting.

**Fig. 5.**
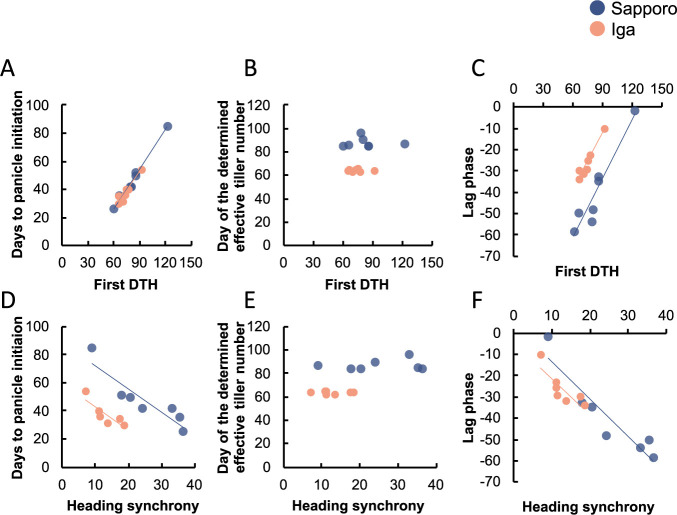
The relationships of first DTH or heading synchrony to the three-lag phase related traits. (A), (B), and (C) show the relationships between first DTH and day to panicle initiation, day of the determined effective tiller number, and lag phase, respectively. (D), (E), and (F) show the relationships between heading synchrony and day to panicle initiation, day of the determined effective tiller number, and lag phase, respectively. Each value represents the average of A58, Kitaake, the four RILs (E-RIL-1, -2 and L-RIL-1, -2), and Koshihikari.

We observed the tiller number transition at seven-day intervals starting 21 days after transplantation. All lines showed a slower increase in tiller number in Sapporo than in Iga (Fig. 4). The slower increase in tiller number at Sapporo resulted in an increase in day of the determined effective tiller number occurring between 84 and 96 days, whereas at Iga, it occurred between 63 and 66 days (Table 2). A good correlation was observed between F-DTH and the days to panicle initiation in each line despite of the two areas (r = 0.99 at Sapporo and r = 0.96 at Iga) (Fig. 5A), whereas F-DTH did not correlate with the day of the determined effective tiller number (Fig. 5B), which was the similar period in each area (Fig. 5B, Table 2). In both areas, panicle initiation occurred earlier in the photo-In varieties (A58, Kitaake, and the four RILs) than in the photo-Se variety, Koshihikari (Table 2). In Sapporo, there was a large difference in panicle initiation among the photo-In varieties, ranging from 25 to 51 days, whereas in Iga, the difference was small, ranging from 30 to 40 days (Table 2). All lines took longer for days to panicle initiation in Sapporo than in Iga except for E-RIL-1 (Table 2). The differences in days to panicle initiation between Sapporo and Iga were less than four days for E-RIL-1 and -2, whereas the differences were more than nine days for L-RIL-1 and -2, and 31 days for Koshihikari (Table 2). These indicated that panicle initiation in Sapporo tended to be delayed in the late heading lines than in the early heading lines. Collectively, the extent of the lag phase was determined by panicle initiation in each line and area (r > 0.97). For example, the lag phase for the photo-In variety Kitaake was –48 days in Sapporo (panicle initiation, 42 days; day of the determined effective tiller number, 90 days) and –32 days in Iga (panicle initiation, 30 days; day of the effective tiller number, 63 days) (Table 2). For the photo-Se variety Koshihikari, the lag phase was negative two days in Sapporo (days to panicle initiation, 85 days; day of the determined effective tiller number, 87 days) and –10 days in Iga (days to panicle initiation, 54 days; day of the determined effective tiller number, 64 days) (Table 2). Taken together, the above results indicated that F-DTH and lag phase are closely related through panicle initiation, which is a determinant of both (Fig. 5A, 5C).

### Correlation analyses for the traits and areas

Pairwise Pearson’s correlation analysis was performed for the photo-In varieties and RILs to determine the relationships between each combination of F-DTH, L-DTH, heading synchrony, Deg_Pre_Hed, and panicle number. The correlation coefficients for the five traits are listed in Table 3. All experimental plots showed negative correlations with F-DTH and heading synchrony and Deg_Pre_Hed (Table 3). F-DTH may be a determining factor for heading synchrony and Deg_Pre_Hed in Sapporo and Iga (Table 3). In addition, positive correlations were observed between the heading synchrony and Deg_Pre_Hed at both locations (Table 3). Although heading synchrony was positively correlated with L-DTH in Iga in 2018, the correlations were less significant and not as consistent as other significant correlations. The low correlations between F-DTH or heading synchrony and panicle number observed in 2018 may not have had major effects on the heading characteristics. In 2019, the relationships between heading properties including days to panicle initiation, day of the determined effective tiller number, and lag phase were analyzed (Table 4). Sapporo and Iga both showed similar correlations for each of the traits related to effective tillers (Table 4). In particular, F-DTH and L-DTH were correlated with days to panicle initiation and lag phase (r > 0.84) (Table 4). In addition, a high correlation was found between days to panicle initiation and lag phase (Table 4). On the other hand, day of the determined effective tiller number did not show a correlation with any of the traits (Table 4). As shown in Fig. 5, F-DTH was positively correlated with days to panicle initiation and lag phase, inversely heading synchrony was negatively correlated with days to panicle initiation and lag phase.

**Fig. 6.**
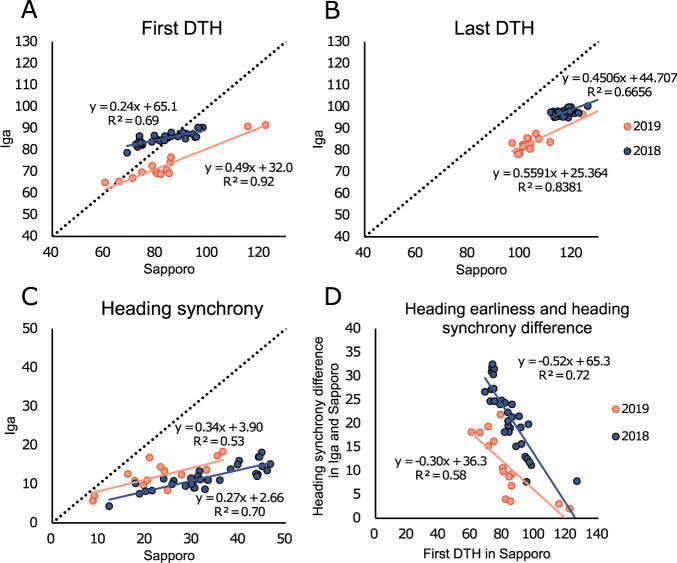
Difference in heading traits between Sapporo and Iga. (A), (B), and (C) show the first DTH, last DTH, and heading synchrony difference between Sapporo and Iga among the lines over two years, respectively. The dotted line represents y = x. (D) Relationship between the heading synchrony difference in the two places and the heading earliness.

To confirm how environmental differences between Sapporo and Iga affected heading traits, we compared heading traits at each site. All heading traits and panicle numbers showed wider variation in Sapporo than in Iga (Fig. 3). The coefficients of determination (r^2^) between Sapporo and Iga for F-DTH, L-DTH, and heading synchrony were greater than 0.53 in the two years (Fig. 6A–6C), and the orders of the heading traits between the lines were mostly consistent in the two areas. The regression lines for F-DTH between Sapporo and Iga exhibited slopes of 0.24 and 0.49 in 2018 and 2019, respectively (Fig. 6A), and the late heading line in 2019 tended to have a greater difference between the two locations. Similar to F-DTH, all lines showed greater heading synchrony in Sapporo than in Iga (Fig. 6C). As heading synchrony was negatively correlated with F-DTHs (Table 3), earlier first heading resulted in larger heading synchrony in Sapporo than in Iga (Fig. 6D). Both F-DTH and heading synchrony among the lines showed large differences in Sapporo, which is located at a high latitude, and the differences were limited to Iga, which is located at a low latitude. As shown in Figs. 3 and 6, variation in heading traits occurred at high latitudes.

### Day-length and temperature variations influenced DTH in photo-In lines

**Fig. 7.**
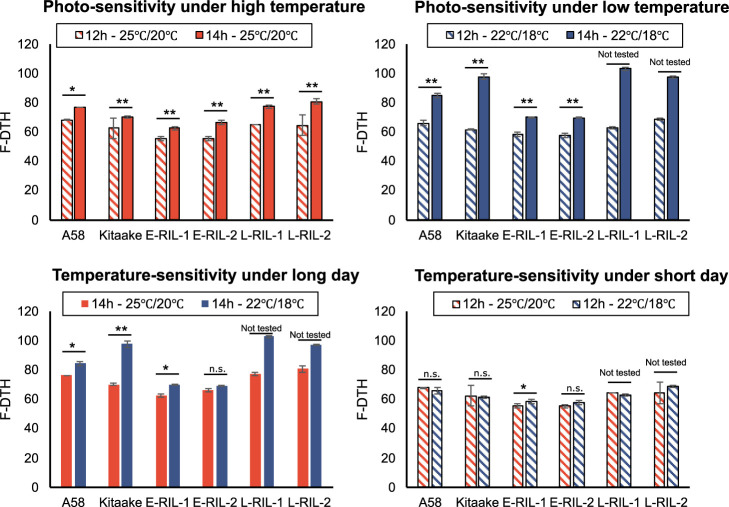
F-DTH of A58, Kitaake, and RILs under four different photoperiod and temperature conditions. Each bar plot indicates the average F-DTH of four plants under four different controlled environments, which comprised combinations of two photoperiod conditions (long day length: 14 h light/10 h dark, short day length: 12 h light/12 h dark) and two temperature conditions (25°C light/20°C dark, 22°C light/18°C dark). Statistical analysis was not performed on some plots because some individuals died.

To validate the differences in the F-DTH of the lines examined in Sapporo and Iga, we tested the heading changes of A58, Kitaake, E-RIL-1, -2 and L-RIL-1, -2 under different day length and temperature conditions: four different environmental conditions were created in growth chambers, consisting of two photoperiod conditions (long day length: 14 h light/10 h dark, short day length: 12 h light/12 h dark) and two temperature conditions (25°C light/20°C dark, 22°C light/18°C dark). Regardless of temperature conditions, DTH was longer under long day lengths than under short day lengths for all lines examined (Fig. 7). In the four lines A58, Kitaake, L-RIL-1, and -2, which had DTHs of 60 days or more under short day lengths, DTHs were affected by long day lengths and low temperatures and were extended by 20 to 40 days, whereas in the two lines E-RIL-1 and -2, which had DTHs of 60 days or less under short day lengths, the DTHs were extended by only approximately 10 days owing to long day lengths and low temperatures (Fig. 7). The observed differences corresponded well with the effects of environmental differences between Sapporo and Iga.

### Genetic variations of heading traits among RILs

To understand heading variation and genetic regulation among photo-In lines, in 2018, we tested 30 RILs derived from a cross between two photo-In varieties, A58 and Kitaake. These photo-In varieties possess non-functional alleles for *Ghd7* and *Osprr37*, which contributed to extremely early heading (Fujino and Sekiguchi 2005a, 2005b, Fujino *et al.* 2013, Nonoue *et al.* 2008, Ota *et al.* 2014), while the contribution of *Hd1* and *DTH8* appeared to be less than that of *Ghd7* and *Osprr37* (Supplemental Table 1). For further analysis in 2019, we evaluated nine photo-In varieties in addition to four RILs, which consisted of two of the earliest and latest heading lines (E-RIL-1, -2 and L-RIL-1, -2). Compared to A58 and Kitaake, these varieties exhibited similar heading patterns, and no significant differences were observed in F-DTH, L-DTH, heading synchrony, or Deg_Pre_Hed in Sapporo either of the year (*t*-test, P > 0.05) (Fig. 3, Supplemental Fig. 1). In Iga, heading synchrony and Deg_Pre_Hed were not significantly different from those of A58 and Kitaake, whereas the F-DTH and L-DTH of Kitaake occurred 2–3 days earlier than those of A58 (Fig. 3, Supplemental Fig. 1). The 30 RILs in 2018 largely segregated each trait from their parents’ values, although their mean values were close to those of their parents (Fig. 3, Supplemental Fig. 1). In 2018, the F-DTH for the 30 RILs ranged from 69 to 98 days and 79 to 90 days in Sapporo and Iga, respectively, whereas the L-DTH ranged from 112 to 126 days and 95 to 100 days, respectively (Fig. 3). L-DTH had a smaller variation than F-DTH (F-test; P < 0.01 in both Sapporo and Iga), and L-DTH showed the weaker correlations between heading synchrony and Deg_Pre_Hed than F-DTH (Table 3). These results suggested that heading synchrony and Deg_Pre_Hed were determined by the timing of the start of heading rather than the end of heading. In Sapporo, heading synchrony for RILs ranged from 18 to 47 days and 18 to 37 days in 2018 and 2019, respectively. In Iga, heading synchrony ranged from 8 to 18 days in 2018 and 11 to 18 days in 2019 (Fig. 3). Deg_Pre_Hed in RILs also showed a distribution very similar to that of heading synchrony; in Sapporo, it was distributed over a wide range from 3 to 19 days in both years, whereas in Iga, it was within 3 days and premature heading rarely occurred. The variations in heading synchrony and Deg_Pre_Hed for the RILs were greater than those for the photo-In varieties. It was also clear that the RILs possessed different genetic factors regulating early heading, heading synchrony, and Deg_Pre_Hed. These traits are more apparent at higher latitudes than at lower latitudes.

### QTL analysis for heading synchrony and F-DTH

To analyze the genetic contributions to heading traits and panicle number, we performed a QTL analysis for RILs at the two locations. Thirty RILs, including E-RILs and L-RILs, were produced using A58 and Kitaake as the parents. The two parental lines have alleles that lack the functions of *Ghd7* and *Osprr37*, which are important for photoperiod sensitivity; therefore, they can essentially induce panicles even on long days. However, in the RILs investigated in Sapporo in 2018, F-DTH varied by more than 30 days. This difference suggests that genetic factors other than the two genes, including F-DTH, affect heading traits. Previously, we obtained 634 SNP markers in A58, Kitaake, and their 30 RILs and identified five QTLs controlling F-DTH by comparing genotype frequencies between the early and late 15 RILs (Koide *et al.* 2019). In this study, we selected 224 SNP markers from 634 polymorphisms that were confirmed based on whole-genome resequencing of A58 and Kitaake. We detected five QTLs for F-DTH, seven for L-DTH, three for heading synchrony, five for Deg_Pre_Hed, and five for panicle number (Table 5, Supplemental Fig. 2). Among the five QTLs for F-DTH, only *qFDTH4* was detected in both Sapporo and Iga, whereas the other three were obtained from Iga. In both Sapporo and Iga, the QTL with the largest effect appeared at the *qFDTH4* locus, accounting for 64% and 41% of the total phenotypic variation in Sapporo and Iga, respectively (Table 5, Supplemental Fig. 2). We detected seven QTLs for L-DTH; however, none were common in Sapporo and Iga. *qLDTH5* explained 40% of the total phenotypic variation in L-DTH (Table 5). We also identified three QTLs for heading synchrony: *qHS1* from Sapporo, *qHS4* from Sapporo and Iga, and *qHS8* from Iga (Table 5, Supplemental Fig. 2). Among these, *qHS4* indicated the largest effect accounted for 63% of the total phenotypic variation in Iga, and the total phenotypic variation in *qHS4* in Sapporo was 27% (Table 5, Supplemental Fig. 2). Five QTLs for Deg_Pre_Hed were detected; *qPH4* was detected in both Sapporo and Iga, and others were detected in either Sapporo or Iga. *qPH4* indicated the largest effect in Sapporo, and *qPH8* indicated the largest effect in Iga. The QTLs, *qDTH4*, *qHS4*, and *qPH4* were mapped to similar positions on chromosome 4 (Table 5). Similarly, *qFDTH8*, *qLDTH8*, *qHS8*, and *qPH8* were located close to each other on chromosome 8 (Table 5). These QTLs were most likely located at the same locus on each chromosome. Multiple trait analysis did not show any epistatic relationships among the QTLs, implying additive effects of these QTLs.

## Discussion

Heading earliness is known to correlate with heading synchrony, and early heading lines grown in high-latitude regions have longer periods of heading synchrony (Ma *et al.* 2009, Takeda 1986). In addition to the relationship between F-DTH and heading synchrony, we found that heading earliness influenced Deg_Pre_Hed, the timing of panicle initiation, and the lag phase (Fig. 5, Table 3). We described significant correlations among four traits, F-DTH, heading synchrony, Deg_Pre_Hed, and lag phase in all test plots (Table 3). The early heading lines with photo insensitivity had larger heading synchrony and Deg_Pre_Hed than the mid- and late-heading lines with photo sensitivity (Fig. 3). We have shown that the long lag phase is also a characteristic of photo-In varieties as determined by panicle initiation, so that panicle initiation not only determines F-DTH but also the extent of the lag phase. Unlike F-DTH, L-DTHs were mostly constant among the lines, and their variations were not large enough to explain the correlation with the variations in heading synchrony, Deg_Pre_Hed, and lag phase for the effective tiller (Fig. 3, Tables 3, 4). Based on these results, we assumed that photo-In varieties exhibited strong apical dominance and that the main stem heading preceded others, resulting in heading asynchrony (Fig. 3). Some studies have been reported that a relationship between heading and tillering, such as a negative relationship between DTH and panicle number in photo-In lines (Fujino 2020) or *Hd3a* promotion of branch formation (Tsuji *et al.* 2015). However, few reports have focused on the heading synchrony and heading order. A detailed understanding of the balance between panicle development and tiller development may contribute to the understanding of F-DTH-related heading characteristics.

Environmental factors also had a significant impact on heading earliness when comparing Sapporo and Iga at different latitudes. All lines had longer periods of heading synchrony and Deg_Pre_Hed in Sapporo than in Iga (Fig. 6C). The differences in heading synchrony between Sapporo and Iga were greater for earlier heading lines and smaller for later heading lines (Fig. 6D). A similar trend was observed in the Deg_Pre_Hed and lag phases, which were well-coordinated with the larger variations in F-DTH in Sapporo than in Iga (Figs. 3, 5, Table 3). Heading earliness showed genetic variation even among photo-In lines. It is clear that heading synchrony and Deg_Pre_Hed are also coordinately affected by F-DTH. Variations in these traits were particularly pronounced in Sapporo, which is located at a higher latitude, and the differences between the photo-In lines were relatively small, even among the photo-In lines in Iga, which is located at a lower latitude. In addition to the genetic variation in early heading, we found that several phenotypic variations, such as heading synchrony, Deg_Pre_Hed, and lag phase, varied greatly depending on the environment.

Focusing on the differences between Sapporo and Iga in terms of the timing of panicle initiation and F-DTH, two characteristics were observed. First, the differences in F-DTH among lines were larger in Sapporo than in Iga, and second, the difference in F-DTH between the two areas was smaller for the early heading lines and larger for the late heading lines. The panicle initiation stage is primarily controlled by day length and temperature (Blümel *et al.* 2015, Preston and Fjellheim 2022). In both years, growing season temperatures were higher in Iga and lower in Sapporo, and day length was shorter in Iga and longer in Sapporo (Fig. 2). To elucidate how environmental differences affect panicle initiation, we evaluated the environmental responses of photo-In lines and four RILs under four different day lengths and temperatures in growth chambers. We observed that DTHs were delayed by low temperatures; however, the DTH differences between temperatures were greater at longer day lengths, and the earlier heading lines showed smaller differences than the later heading lines (Fig. 7). This result explains the difference in F-DTH between the two paddy fields, indicating that the difference resulting from the effect of temperature on heading was more pronounced in Sapporo than in Iga, owing to the long day length and low temperature. We previously reported that photo-In varieties showed limited low-temperature-mediated delays in heading due to an extremely short photo-Se phase (Sakaguchi *et al.* 2024), which is a subphase of the vegetative growth phase of rice (Vergara and Chang 1985). This study, conducted in paddy fields at different latitudes, demonstrated that F-DTH in rice responded to day length and temperature and affected heading synchrony, Deg_Pre_Hed, and lag phase differentially. We also found that the earlier-heading lines exhibited premature heading in Sapporo but not in Iga. Premature heading has been reported to be induced by a long seedling period and high temperatures during the seedling stage (Hu *et al.* 2015, Osada *et al.* 1975, Sakata *et al.* 2004). In the present study, the seedling growth period was longer in Sapporo in both years (Table 1). Although the temperatures during seedling growth remained low, a longer seedling growth period resulted in higher accumulated temperatures until transplanting (Fig. 2). Environmental conditions, long day length, and low temperature in Sapporo caused long period of heading synchrony and premature heading in photo-In lines. This phenomenon may be associated with an extremely shortened photoperiod-sensitive phase, which is a characteristic of photo-In lines (Sakaguchi *et al.* 2024).

The increase in tillers at Sapporo was slower than that at Iga, which resulted in an increase in the day of the determined effective tiller number (Fig. 4, Table 2). The growth of tillers is regulated by several factors, such as solar radiation, field water, and nitrogen supply (Yuan *et al.* 2024). However, in high-latitude regions, temperature is considered to be the most important factor. Indeed, the temperature in Sapporo was relatively low compared to that in Iga, particularly during the early stages of transplanting (Fig. 2). This could have contributed to the delayed tiller growth. Given that there was no correlation between F-DTH and tillering ability (tiller growth rate or day of the determined effective tiller number), it can be inferred that this tillering delay in Sapporo was attributed to extended heading synchrony and the lag phase, particularly for the early heading lines. There is a dilemma between the two opposing issues in rice cultivation in high-latitude regions such as Hokkaido: early heading accompanied by low synchronous heading and high premature heading can prevent simultaneous cold damage, while late tillers are exposed to cold stress (Takeda 1986). If the genetic basis of the relationship between early heading and asynchronous heading in high-latitude varieties is elucidated, it may be possible to effectively control these traits.

While there is substantial research on the process of heading itself and the underlying mechanisms have been elucidated in considerable detail (Doi *et al.* 2004, Izawa *et al.* 2000, Koo *et al.* 2013, Matsubara *et al.* 2008, 2012, Yano *et al.* 2000), there is a paucity of reports on the genetic characteristics of traits related to heading. Furthermore, there is a dearth of knowledge concerning traits, such as heading synchrony, premature heading, and effective tillers. Ishimaru and Kashiwagi (2004) and Ma *et al.* (2009) described genetic differences in heading synchrony between *japonica* and *indica* rice. However, there have been no reports of genetic variations in *japonica*. In this study, we analyzed the RILs derived from a cross between the two *japonica* varieties, A58 and Kitaake (Table 3). We detected five QTLs involved in F-DTH, seven involved in L-DTH, three involved in heading synchrony, five involved in Deg_Pre_Hed, and five involved in panicle number (Table 5). Koide *et al.* (2019) previously reported that these RILs indicated five QTLs with different effects on DTH. Among the five QTLs one located in chromosomes 4 could be present close to *qFDTH4*, which had the largest effect on DTH. Closer to *qDTH4*, *qHS4* and *qPH4* were mapped and these QTLs detected in both Sapporo and Iga. The three QTLs showed the largest effects among the QTLs for F-DTH, heading synchrony, and degree of premature heading in Sapporo, suggesting that the QTLs had effects not only on F-DTH, but also on heading synchrony and effective tillering. Similarly, *qFDTH8*, *qLDTH8*, and *qHS8* were closely mapped and may be associated with multiple heading traits. No major heading genes were located on QTLs on chromosomes 4 and 8 and unknown genes may be present on these QTL regions. Owing to the lack of detection of epistatic interactions between QTLs located on chromosomes 4 and 8, these QTLs had additive effects on F-DTH, heading synchrony, and effective tillers. However, other QTLs were detected in either F-DTH, L-DTH, or heading synchrony, and may be able to control F-DTH or heading synchrony without affecting the other. Some QTLs associated with heading synchrony were reported by Ishimaru and Kashiwagi (2004) and Ma *et al.* (2009), however, we found QTLs for heading synchrony in different regions. We expect that these hidden QTLs will resolve the dilemma of early heading in photo-In lines, which entails the generation of photo-In lines that exhibit early heading, high heading synchrony, low premature heading, and high proportion of effective tillers.

Since premature heading is a trait that is strongly affected by temperature, the QTL found in this study may be involved in temperature sensitivity. *Ghd7* has been reported as a temperature-sensitive gene in rice, whose expression is known to be decreased under high temperature conditions, leading to increased expression level of the florigen gene, *RFT1* (Nagalla *et al.* 2021). As shown in Fig. 7, under the long day length, DTH showed different temperature responses among the lines even with the non-functional *Ghd7*. Future analyses are needed to clarify how *Ghd7* is involved in temperature sensitivity in photo-In varieties, in which *Ghd7* is non-functional.

Avoiding stress by changing the heading period is considered an effective way to cope with the extreme weather conditions caused by intense heat in summer. We confirmed that the photo-In varieties showed early heading in low- and high-latitude regions. Furthermore, our findings revealed that photo-In varieties cultivated in low-latitude regions demonstrated the requisite characteristics for utilization. These varieties exhibited a range of heading synchrony comparable to that observed in photo-Se varieties at high latitudes, along with a relatively low frequency of premature heading. The heading traits of the photo-In varieties cultivated in low-latitude areas may contribute to preventing damage to rice growth, particularly that caused by high temperatures, through early rice cultivation without reducing effective tiller numbers. In Japan and temperate regions of the Northern Hemisphere, the use of photo-In varieties permits the commencement of sowing and planting from the end of February to early March, and the completion of harvesting processes before July.

## Author Contribution Statement

S.S., Y.O., and Y.K. designed the research plans. S.S. and Y.O. performed the trait analyses at Sapporo and Iga, respectively. S.S., Y.O., and M.I.H. performed the QTL analyses. S.S. and Y.K. prepared the manuscript and wrote the article. Y.O. and M.I.H. confirmed the detailed manuscript structure. The reason why this paper is presented by the two corresponding authors, Y.O. and Y.K., is that the experiments were basically performed independently in Iga and Sapporo with the same materials, each of which had responsibility.

## Supplementary Material

Supplemental Figures

Supplemental Table

## Figures and Tables

**Table 1.** Test environments and materials used in this study

Year	Place	Date of sowing	Date of transplanting	Materials
2018	Iga	19-Apr	10-May	A58, Kitaake, Koshihikari, 30 RILs*^a^*
	Sapporo	6-May	7-Jun	A58, Kitaake, Koshihikari, 30 RILs
2019	Iga	24-Apr	15-May	9 photo-In varieties*^b^*, Koshihikari, Mie 33, 4 RILs
	Sapporo	9-May	4-Jun	9 photo-In varieties, Koshihikari, Mie 33, 4 RILs

*^a^* RILs were derived from the cross between A58 and Kittake, and each genotype is shown in Supplemental Table 1. *^b^* Nine photo-In varieties were A58, Kitaake, Akage, Norin 20, Hakuchomochi, Kirara 397, Hoshinoyume, Nanatsuboshi, and Daichinohoshi.

**Table 2.** The difference in days to panicle initiation, day of the determined effective tiller number, and lag phase in Sapporo and Iga.

	Days to panicle initiation (A)				Day of the determined effective tiller number (B)				Lag phase (A–B)*^a^*	
	Sapporo	Iga			Sapporo	Iga			Sapporo	Iga
A58	42.0 ± 2.3	36.1 ± 0.2	**		96 ± 2.3	65 ± 2.1	**		–54.0	–29.3
Kitaake	42.0 ± 1.2	31.5 ± 0.3	**		90 ± 2.6	63 ± 0.0	**		–48.2	–31.5
Early RIL-1	25.7 ± 0.6	30.3 ± 0.2	**		84 ± 4.3	64 ± 1.1	**		–58.7	–33.9
Early RIL-2	35.3 ± 0.7	35.0 ± 0.3	n.s.		86 ± 2.7	65 ± 1.5	**		–50.2	–29.8
Late RIL-1	51.5 ± 1.6	40.4 ± 0.3	**		84 ± 1.3	66 ± 1.5	**		–32.8	–25.4
Late RIL-2	49.4 ± 1.0	40.3 ± 0.4	**		84 ± 2.7	63 ± 0.0	**		–35.0	–22.7
Koshihikari	84.9 ± 0.4	54.2 ± 0.1	**		87 ± 3.2	64 ± 1.1	**		–1.8	–10.0

*^a^* Lag phase was calculated by days to panicle initiation minus day of the determined effective tiller number. ^b^ Astarisks indicate the statistical difference between Sapporo and Iga.

**Table 3.** Pairwise Pearson correlation coefficients among heading characteristics

Year	Place	Traits	First DTH	Last DTH	Heading synchrony	Deg_Pre_Hed	Panicle number
2018	Sapporo	First DTH	1.00				
		Last DTH	0.18	1.00			
		Heading synchrony	–0.93**	0.18	1.00		
		Deg_Pre_Hed	–0.90**	–0.10	0.87**	1.00	
		Panicle number	0.49**	0.34	–0.37*	–0.33	1.00
	Iga	First DTH	1.00				
		Last DTH	0.07	1.00			
		Heading synchrony	–0.87**	0.42*	1.00		
		Deg_Pre_Hed	–0.75**	0.26	0.81**	1.00	
		Panicle number	0.43*	0.07	–0.35*	–0.29	1.00
2019	Sapporo	First DTH	1.00				
		Last DTH	0.52	1.00			
		Heading synchrony	–0.87**	–0.04	1.00		
		Deg_Pre_Hed	–0.95**	–0.41	0.88**	1.00	
		Panicle number	0.65*	0.77**	–0.31	–0.61*	1.00
	Iga	First DTH	1.00				
		Last DTH	0.55	1.00			
		Heading synchrony	–0.60*	0.34	1.00		
		Deg_Pre_Hed	–0.66*	–0.44	0.69**	1.00	
		Panicle number	0.44	0.72**	0.19	–0.44	1.00

In 2018, two photo-In varieties and 30 RILs were tested. In 2019, nine photo-In varieties and four RILs were tested.

**Table 4.** Pairwise Pearson correlation coefficients between traits related to lag phase

Place	Traits	Days to panicle initiation	Day of the determined effective tiller number	Lag phase
Sapporo	First DTH	0.99**	0.02	0.95**
	Last DTH	0.93**	0.24	0.84*
	Heading synchrony	–0.91**	0.22	–0.93**
	Degree of premature heading	0.55*	0.67	0.38
	Panicle number	–0.77	–0.22	–0.69
	Days to panicle initiation	1.00	–0.04	0.97**
	Day of the determined effective tiller number		1.00	–0.26
	Lag phase			1.00
Iga	First DTH	0.96**	–0.06	0.97**
	Last DTH	0.95**	–0.12	0.97**
	Heading synchrony	–0.84*	–0.04	–0.83*
	Degree of premature heading	0.18	–0.23	0.21
	Panicle number	–0.45	0.21	–0.48
	Days to panicle initiation	1.00	0.09	0.99**
	Day of the determined effective tiller number		1.00	–0.05
	Lag phase			1.00

**Table 5.** Overview of QTL*^a^* identified for all traits in Sapporo and Iga

Trait	QTL		Place	Chromosome	Peak position (cM)	LOD score	Variance explained of total (%)	Additive effect*^b^*	LOD threshold
First DTH	*qFDTH2*		Iga	2	47.7	3.4	9	1.0	2.9
	*qFDTH4*	*	Sapporo	4	120.8	12.4	64	7.8	3.7
		*	Iga	4	120.8	8.4	41	1.8	2.9
	*qFDTH8*		Iga	8	55.2	5.0	20	–1.3	2.9
	*qFDTH11*		Iga	11	0.0	4.5	14	1.0	2.9
	*qFDTH12*		Iga	12	17.2	4.0	13	–1.1	2.9
Last DTH	*qLDTH2*		Iga	2	75.9	2.9	16	–0.7	2.8
	*qLDTH3*		Sapporo	3	2.1	3.6	36	2.1	2.9
	*qLDTH4*		Iga	4	55.8	5.0	28	–0.8	2.8
	*qLDTH5*	*	Sapporo	5	81.8	4.7	40	–2.3	2.9
	*qLDTH6*		Sapporo	6	2.2	4.0	26	–1.8	2.9
	*qLDTH8*		Iga	8	62.4	5.1	35	0.9	2.8
	*qLDTH9*		Iga	9	196.5	5.1	31	0.9	2.8
Heading synchrony	*qHS1*		Sapporo	1	111.8	5.6	18	4.0	3.4
	*qHS4*		Sapporo	4	120.8	7.4	27	–5.3	3.4
		*	Iga	4	120.8	11.5	63	–2.6	3.3
	*qHS8*		Iga	8	62.4	7.7	23	1.4	3.3
Degree of premature heading	*qPH4*		Sapporo	4	122	7.5	39	–2.7	3.7
			Iga	4	123	3.7	19	–0.6	2.2
	*qPH5*		Sapporo	5	19	4.9	24	1.9	3.7
	*qPH8*	*	Iga	8	64	7.0	45	0.9	2.2
	*qPH12-1*		Iga	12	69	3.8	23	1.0	2.2
	*qPH12-2*		Iga	12	82	2.5	12	–0.7	2.2
Panicle number	*qPN1*		Sapporo	1	58	3.2	19	–1.9	2.7
	*qPN2*		Sapporo	2	35	2.8	18	1.8	2.7
	*qPN9*		Iga	9	28	3.4	23	–1.4	2.9
	*qPN11*		Sapporo	11	56	3.6	24	–2.2	2.7
			Iga	11	43	4.3	25	–1.3	2.9
	*qPN12*		Iga	12	39	3.9	30	–1.6	2.9

*^a^* QTLs that explain more than 40% of the total variance are marked with an asterisk. *^b^* Additive effects of A58 allele.
