# Supplementary material for: Genetic and environmental regulation of early heading in photoperiod-insensitive : rice: impacts on heading synchrony, premature heading, and tiller development lag
Source: Breed Sci. 2025 Jul 31;75(4):267–80. doi: 10.1270/jsbbs.25002 (PMC13051634; doi:10.1270/jsbbs.25002)
Supplement: Supplementary file 1 — Supplemental Figures [file 75_267_s1.pdf]

doi: 10.1270/jsbbs.25002

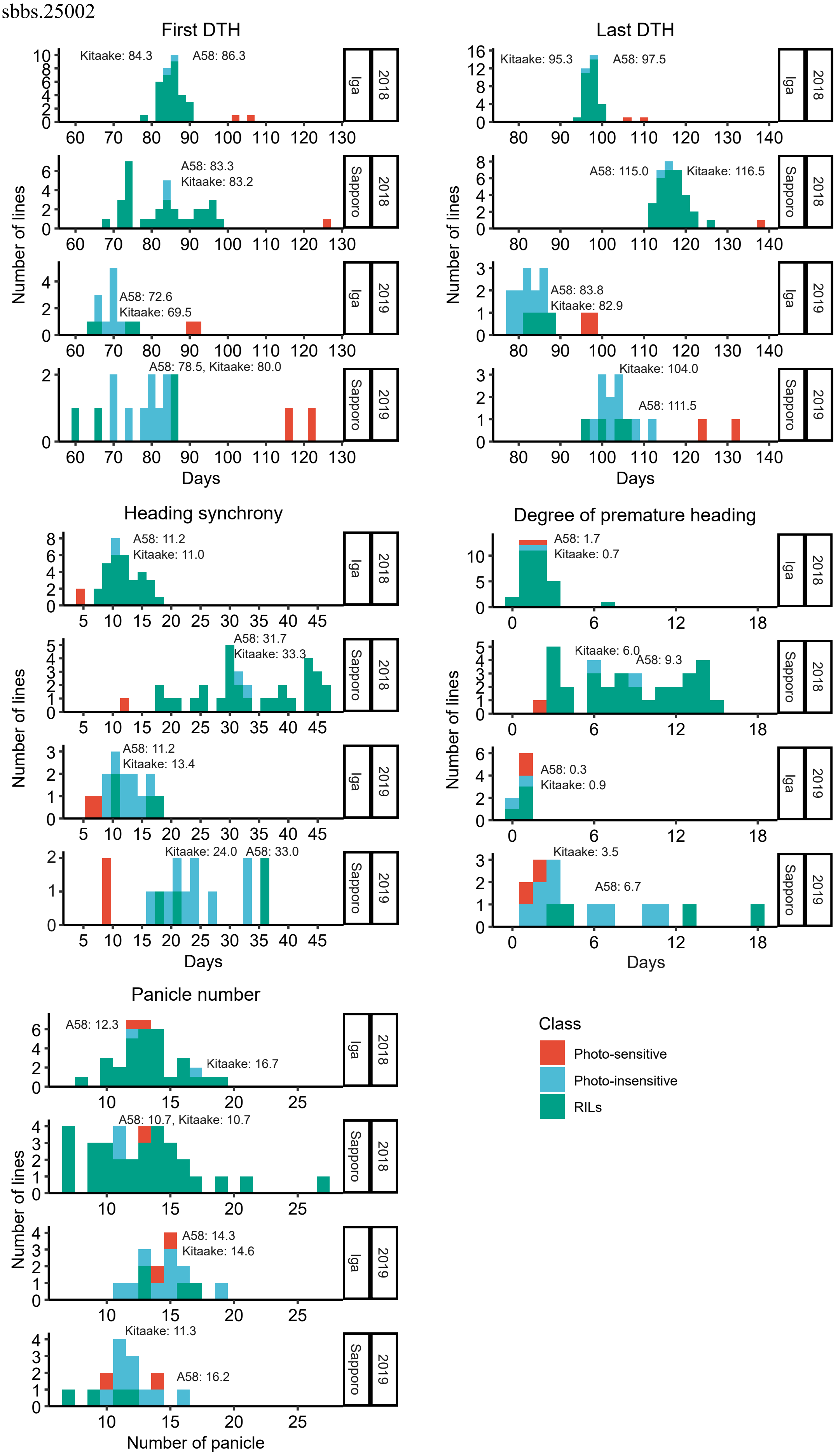

Supplemental Fig. 1. Frequency distribution of heading traits and panicle number.
Photo-Se varieties, photo-In varieties, and RIL distribution are shown in different colors. In 2018, two photo-In varieties, one photo-Se variety, and 30 RILs were used. In 2019, nine photo-In varieties, two photo-Se varieties, and four RILs (E-RIL-1, -2 and L-RIL-1, -2) were used. In Iga in 2019, degree of premature heading was measured for two out of nine photo-In varieties.

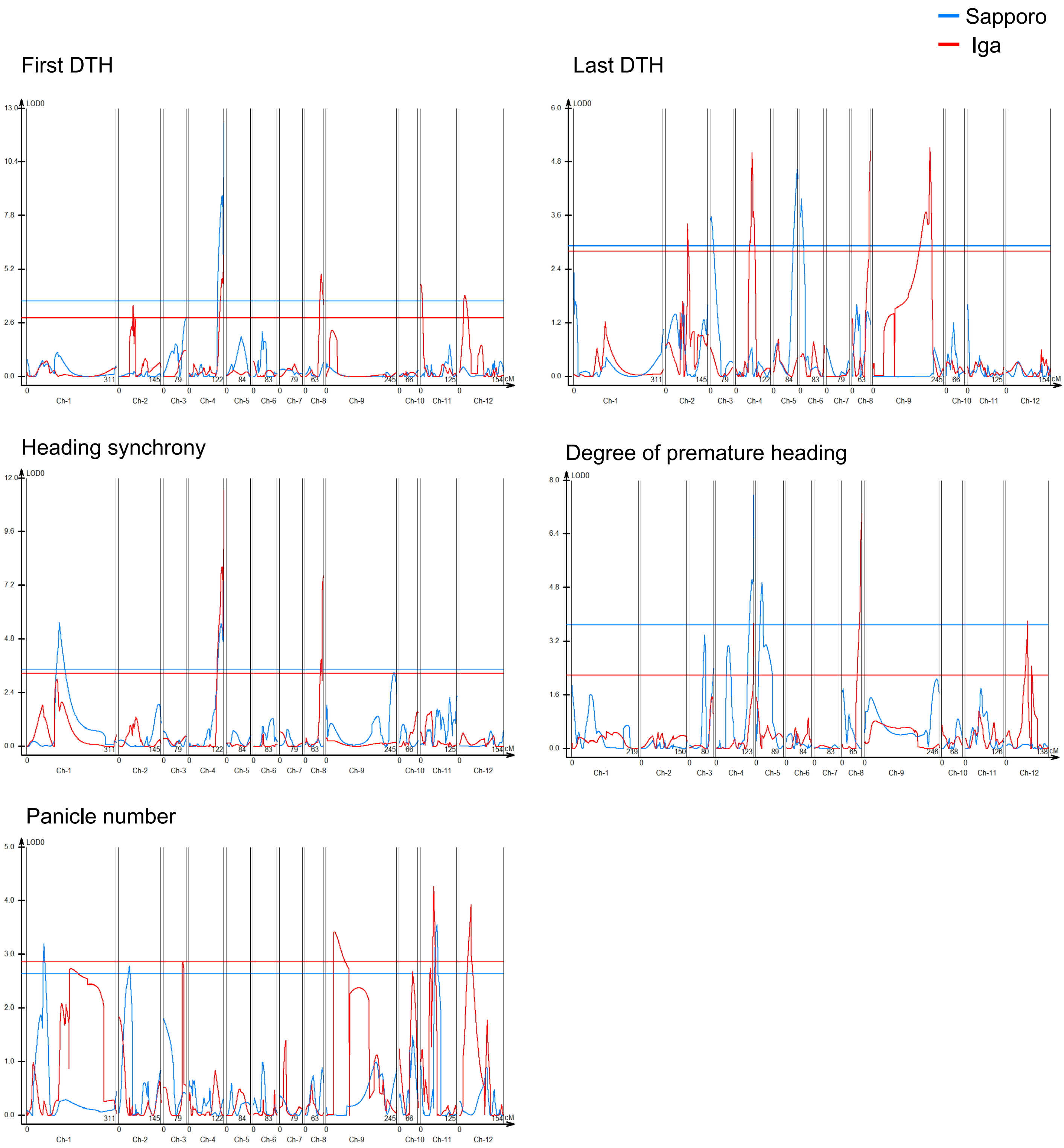

Supplemental Fig. 2. Likelihood odds ratio (LOD) score of qualitative trait loci (QTL)s of F-DTH, L-DTH, heading synchrony, degree of premature heading and panicle number.
Four QTLs for the first DTH, seven for the last DTH, three for heading synchrony, five for degree of premature heading, and five for panicle number were detected in 30 RILs derived from A58 and Kitaake, with 224 SNP markers. The LOD scores for Sapporo and Iga are shown in different colors. The horizontal line shows the threshold determined by 1000 permutation tests.
